# Delocalized spin states at zigzag termini of armchair graphene nanoribbon

**DOI:** 10.1038/s41598-024-62624-9

**Published:** 2024-05-21

**Authors:** Stefan Šćepanović, Amina Kimouche, Jovan Mirković, Gehad Sadiek, Tillmann Klamroth, Abdou Hassanien

**Affiliations:** 1 Jozef Stefan Institute, 39 Jamova, 1000 Ljubljana, Slovenia; 2 Faculty of Sciences, University of Montenegro, 81000 Podgorica, Montenegro; 3 Institute of Physics and Astronomy, University of Potsdam, 14476 Potsdam, Germany; 4 Department of Applied Physics and Astronomy, University of Sharjah, 27272, Sharjah, UAE; 5 Institute of Chemistry, University of Potsdam, 14476 Potsdam, Germany

**Keywords:** Graphene nanoribbon, Spin states, Kondo effect, Spin–spin correlations, Nanoscience and technology, Physics

## Abstract

Using scanning tunneling microscopy and spectroscopy we demonstrate a revival of magnetism in 7-armchair nanoribbon by unpassivated atoms at the termini. Namely, a pair of intense Kondo resonances emerges at the peripheries of zigzag terminus revealing the many-body screening effects of local magnetic moments. Although Kondo resonance originates from a missing local orbital, it extends to a distance of 2.5 nm along the edge of the ribbon. The results are complemented by density functional theory calculations which suggest a possible coupling between Kondo states despite screening effects of substrate electrons. These findings indicate a possibility to restore intrinsic magnetic ordering in graphene nanoribbon without major structural modifications.

## Introduction

There has been a flurry of research activities to induce magnetism in carbon nanostructures^1^ serving as key elements for graphene-based spintronic and quantum devices^2–6^. These systems with various structural topologies utilize advanced techniques of surface chemistry^7^ to engineer intrinsic π-magnetism^8^ based on Lieb’s theorem of bipartite lattice^9^ and Ovchinnikov’s rule. Moreover, the work has been expanded further to induce topological frustration of the π-electron network^10–14^ and/or a polarization of low-energy states^15–17^. Accordingly, several research groups have found conceptually new ways to engineer a plethora of properties combining magnetism with charge transport for advanced spintronic devices^6,10,18^. The main drive for this is that atomically clean GNRs offer very low spin relaxation and decoherence channels due to small spin–orbit and hyperfine couplings^19^. Beside the large spin correlation length^6^, the small spin–orbit coupling can be utilized to introduce desirable topological effects at the edges of extended graphene^20^. Furthermore, edge states are naturally gapped from bulk states, especially in semiconducting ribbons^3^. However, as edge states are always passivated via atom abstraction and radical recombination, the overall ground state remains nonmagnetic. In an attempt to work around this issue, early works have shown that removing a carbon atom^21^, substitutional doping^22^ or locally forming *sp*^3^ orbitals by adding a hydrogen atom to the graphene lattice^23^, generates a delocalized magnetic moment. Further work has also shown that intramolecular junction of chiral graphene nanoribbon can occasionally host spin states^23^. However, the occurrence of such states is rather uncontrolled due to the nature of the self-assembled molecular junctions. Interestingly, the study has shown that spin ordering can be initiated by extracting H atom with the tip of scanning tunneling microscope (STM). Through engineering a sublattice imbalance in graphene nanoribbon lattice^9^, a different strategy was employed. In their work, Sun et al.^24^ have used the imbalance between A and B sublattices to successfully initiate spin states with zigzag decorated edges of 7-armchair ribbon. In this way, several magnetic phases have been obtained. Despite this pioneering work, the type of long-range magnetic ordering remained elusive due to the difficulty of precise control over the locations and the orientations of the imbalanced sublattices.

In a remarkable step, Ruffieux et al.^25^ have successfully fabricated atomically precise zigzag GNR on Au(111) which was predicted earlier to host spin polarized states^4^. However, such a passivated ribbon also has a small band gap to withstand interaction effects from bulk states^26^ and hybridization with substrate which cause magnetic states to quench. For this reason, it is imperative to test magnetism of zigzag edges in semiconducting ribbons. With that in mind, Wang et al.^27^ have confirmed the predicted energy splitting of 1.9 eV at the zigzag termini of 7-armchair GNR on single layer of *NaCl*. Xu et al.^28^ have fabricated ribbons with non-planar zigzag termini on Au(111) substrate. The decorated spin sites are engineered by altering the atomic arrangement at the nanoribbon’s edges. However, the structure of the ribbon is not well-defined making it difficult to assert the location of the resulting signatures of Kondo states.

In this work we show a rather simple reductionist strategy to introduce spin states at the termini of armchair nanoribbon by removing the attached hydrogen atoms. As zigzag atoms are more reactive, it is expected that hydrogen atoms might be released during the annealing process. Using scanning tunneling microscopy and spectroscopy, we observe intense and delocalized zero bias peaks near the ribbon termini. Temperature dependence of half width at half maximum confirms that the peak is due to Kondo effect emanating from the many body interactions of edge π electrons with a local magnetic moment. Remarkably, the magnetic states are strongly visible, even though the ribbon is interacting with Au(111) substrate, and propagate to a distance of 2.5 nm from the termini, thereby outfacing the dephasing effects. These magnetic states often come in pairs that are located at the peripheries of ribbon termini with a weaker but non-vanishing intensity along their line profile. This may indicate more complex spin structures mediated by magnetic tails interactions with a possible spin polarization along the armchair sides of the ribbon.

## Results

**Figure 1:**
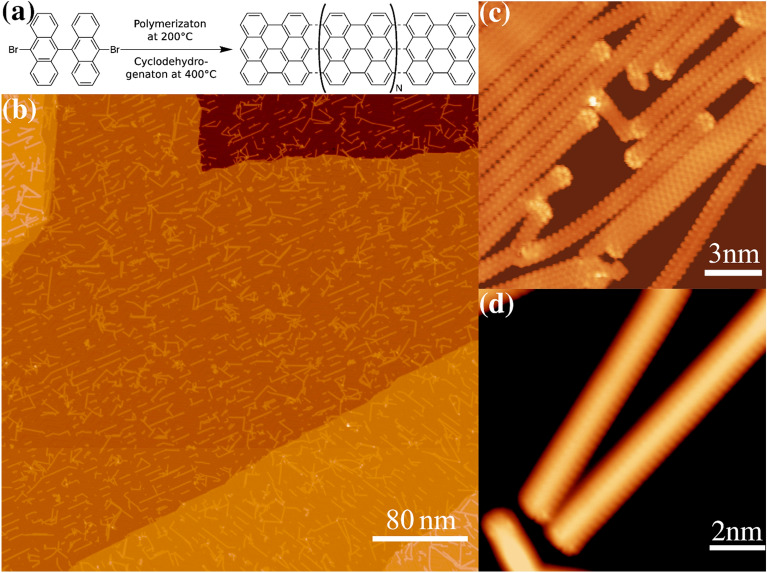
Typical example of clean GNR over Au(111). (**a**) is a schematic overview of surface assisted synthesis of GNR from the DBBA precursor. (**b**) is a large scale STM topographic image showing well isolated ribbons of various lengths which gave the opportunity to access the electronic properties of isolated GNR especially near the termini. (**c**) High resolution image of dense sample showing the charge density modulation on the armchair ribbons and how they fuse to form larger ribbons in units of 7 atoms wide. The ripple represents the charge density modulation with a period equals half Fermi wavelength of 7.5 Å. In all GNRs, the charge density modulations of the edge states have spatially unique asymmetric pattern compared to bulk states electrons. Bias parameters are − 315 mV and − 30 pA. (**d**) High resolution image on two ribbons with isolated edges. Bias parameters are − 500 meV and 20 pA. All images were taken at 4.2 K. The images were treated minimally for contrast enhancement.

The procedures for the on-surface synthesis of GNR from DBBA precursor are shown in Fig. 1a. This usually leads to good quality samples with various coverage depending on the period of the evaporation. Figure 1b shows a typical topographic image of isolated AGNR which are epitaxially oriented along the high symmetry axes of Au(111). The ribbons are synthesized with various lengths ranging from 3 to 40 nm which gives the opportunity to laterally and/or vertically manipulate single ribbon by STM tip. Interestingly, the ribbon can be easily manipulated with relatively mild tunneling parameters (− 50 mV and 3 nA) indicating a weak attachment with the underlying substrate. We have also noticed, that after post annealing process the number of self-assembled junctions has increased, which indicates enhanced reactivity of edges as a result of hydrogen abstraction. Similar behavior of self-assembled zigzag edges has been observed in extended traingulene structures and was attributed to the enhanced zigzag edge reactivity by the presence of unpaired electrons^29^. Nevertheless, we have also observed isolated individual GNRs which gave us the opportunity to study the intrinsic electronic properties at GNR terminus.

Figure 1c represents a typical example of denser sample showing atomically clean termini with a higher contrast due to scattering effects. The bended GNR demonstrates a weak bonding with the underlying substrate as well as flexibility to accommodate lattice mismatch or steric atomic constriction. A close up view on two isolated ribbons is shown in Fig. 1d. The interference patterns are due to coherent scattering of electron states as they bounce off from termini and edges^30^. Its occurrence with relatively large bias windows of − 500 meV highlights a constant phase relationship of electrons as they tunnel across edges without any appreciable dephasing effects from bulk states. This interesting characteristic of phase coherence on edge state can be further demonstrated by mapping interaction effects at the edges and terminus of spin active GNR. Generally speaking, the simplest approach to induce spin active site on GNR is by hydrogen abstraction as it will lead to open-shell structure of edge π band. The possibility of successful hydrogen abstraction at the terminus or any other locations can be tested by mapping the low bias scanning tunneling spectroscopy (STS)^23^.

## Discussion

**Figure 2:**
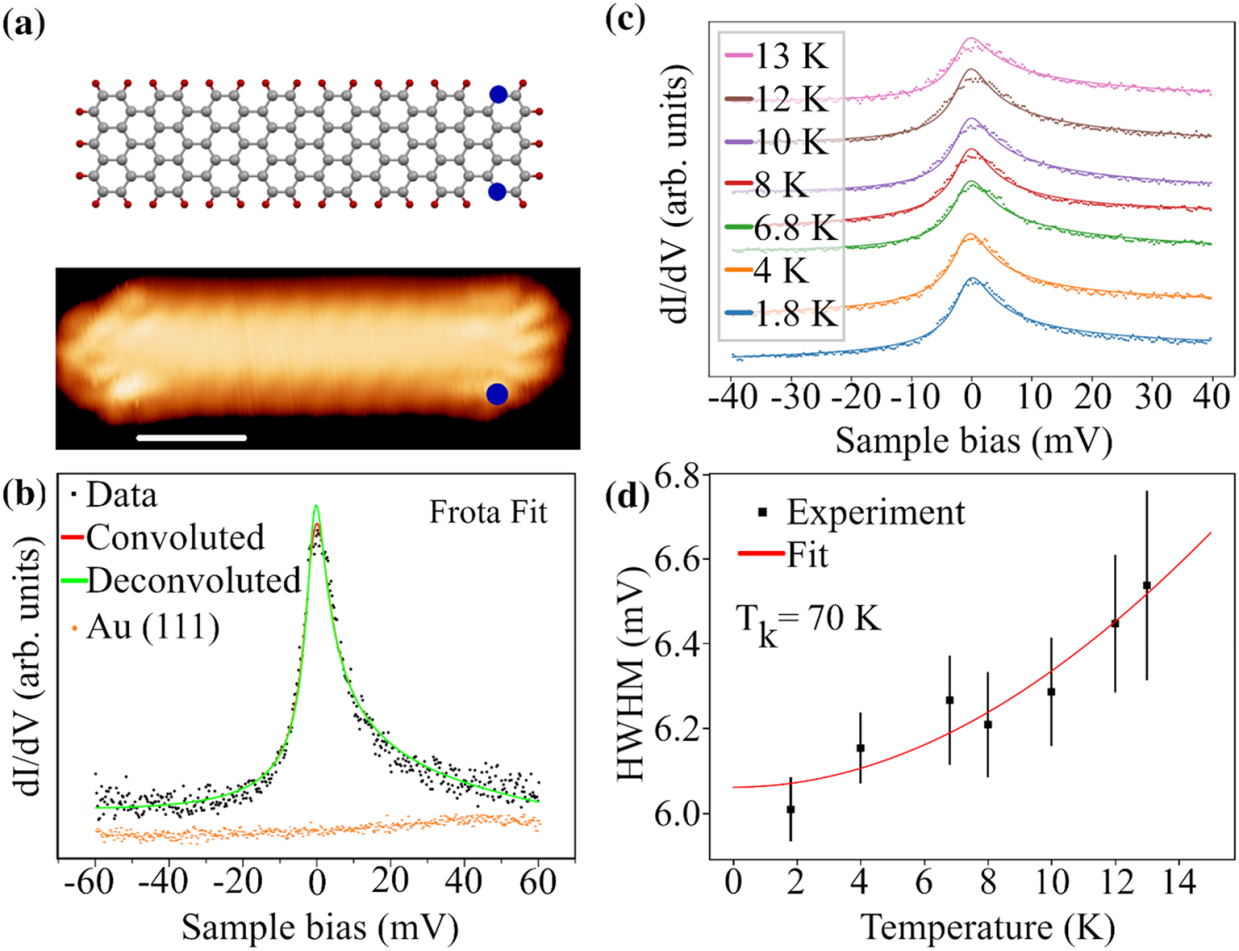
Kondo resonance at the zigzag terminus of GNR**.** The upper panel in (**a**) show the structure of GNR with unpassivated site to mark position where Kondo resonance is the most intense. The lower panel shows a topographic STM image of an isolated GNR. The blue dot points at the position where dI/dV was recorded at various temperatures. The scale bar is 1 nm. (**b**) A typical example of dI/dV spectrum shows asymmetric ZBP. The spectrum was deconvoluted (green curve) from the lock-in signal as well as the thermal broadening to obtain the intrinsic width of Kondo resonance^31^. Finally, the experimental data are fitted to Fano-Frota spectral function to yield HWHM of 6.1 meV. Set point: 1 nA, 100 mV. Lock-in: 733 Hz, 0.6 mV. (**c**) The temperature dependence of Kondo resonance is recorded at the same location. (**d**) The temperature dependence fit to Fermi-liquid formula yields a Kondo temperature of 68 K.

In the following sections we shall focus on the zigzag termini to study the emergent spin state as a result of unpassivated sites. Figure 2a shows an example of a short ribbon of size 3.5 nm. The model shows the lattice structure of (7, 16) with two unpassivated sites marked in blue dots near the zigzag terminus. The locations of these active sites are determined from scanning tunneling spectroscopy (STS) profile near the terminus. Although it is very difficult to determine precisely which atom is unpassivated near the terminus, these assignments agree very well with density function theory calculations as the most stable structure configurations, as we shall see in the following sections. The most characteristic feature of STS at zigzag termini is the presence of asymmetric peak located at zero bias with a range of ~ 2.5 nm along the armchair edge.

The variation in the local density of states ($\Delta$ LDOS) due to the presence of magnetic sites can be described by either Fano-Frota or Fano-Lorentz line-shapes^32^. Unless otherwise stated, we have mostly used Fano-Frota function to fit our experimental data, namely:

1 $$\Delta LDOS\left(E\right)\propto \mathfrak{I}\left[i{e}^{i{\phi }_{q}}\sqrt{\frac{i{\Gamma }_{F}}{E-{E}_{K}+i{\Gamma }_{F}}}\right]$$

E_K_ is the position of the Kondo resonance and ${\phi }_{q}$ is a form factor, which determines the line shape as a function of position from the magnetic site. The change in the line shape as a function of position is qualitatively captured by this form factor. ${\Delta }_{F}=$ 2.542 Г_F_ is Frota line half width at half maximum (HWHM) of the Kondo resonance, which gives an estimate to Kondo temperature T_K_ by the following formula: Г_F_ = 1.43 K_B_T_k_^33^. The fitting function of Eq. (1) reproduces the shape of the Kondo resonance very well, both at various temperatures and positions.

Unlike the well-known phonon dressed Tamm states which usually appear at passivated zigzag termini^34^ or the zero energy charged states^24^, the zero bias peak (ZBP) is much sharper with no appreciable shift in energy due to charging effects^24^. The relatively large spatial range of the ZBP indicates that it may originate from an open-shell π orbital. Furthermore, the shape of the resonance and the asymmetric changes of the peak profile along the edge of the GNR are perfectly captured by Fano-Frota line shape^32,35^ (see supplementary information). In order to assert that the ZBP is due to Kondo resonance, we studied the temperature dependence of Kondo resonance. Results are shown in Fig. 2c where solid curves represent the fitting of deconvoluted Fano-Frota spectral function to determine the intrinsic value for the HWHM. To determine Kondo temperature, we fit the HWHM value to the following Fermi liquid formula:

2 $$HWHM=\Gamma =\frac{1}{2}\sqrt{{\left(2\alpha {K}_{B}T\right)}^{2}+{\left(2{K}_{B}{T}_{K}\right)}^{2}}$$

As shown in Fig. 2d, the temperature dependence displays a good fit with Eq. (2) and gives a value of 68 K for the Kondo temperature and 4.07 for α. Accordingly, the observed temperature dependence can be explained as a result of electron–electron scattering in the Kondo regime^36^.

**Figure 3:**
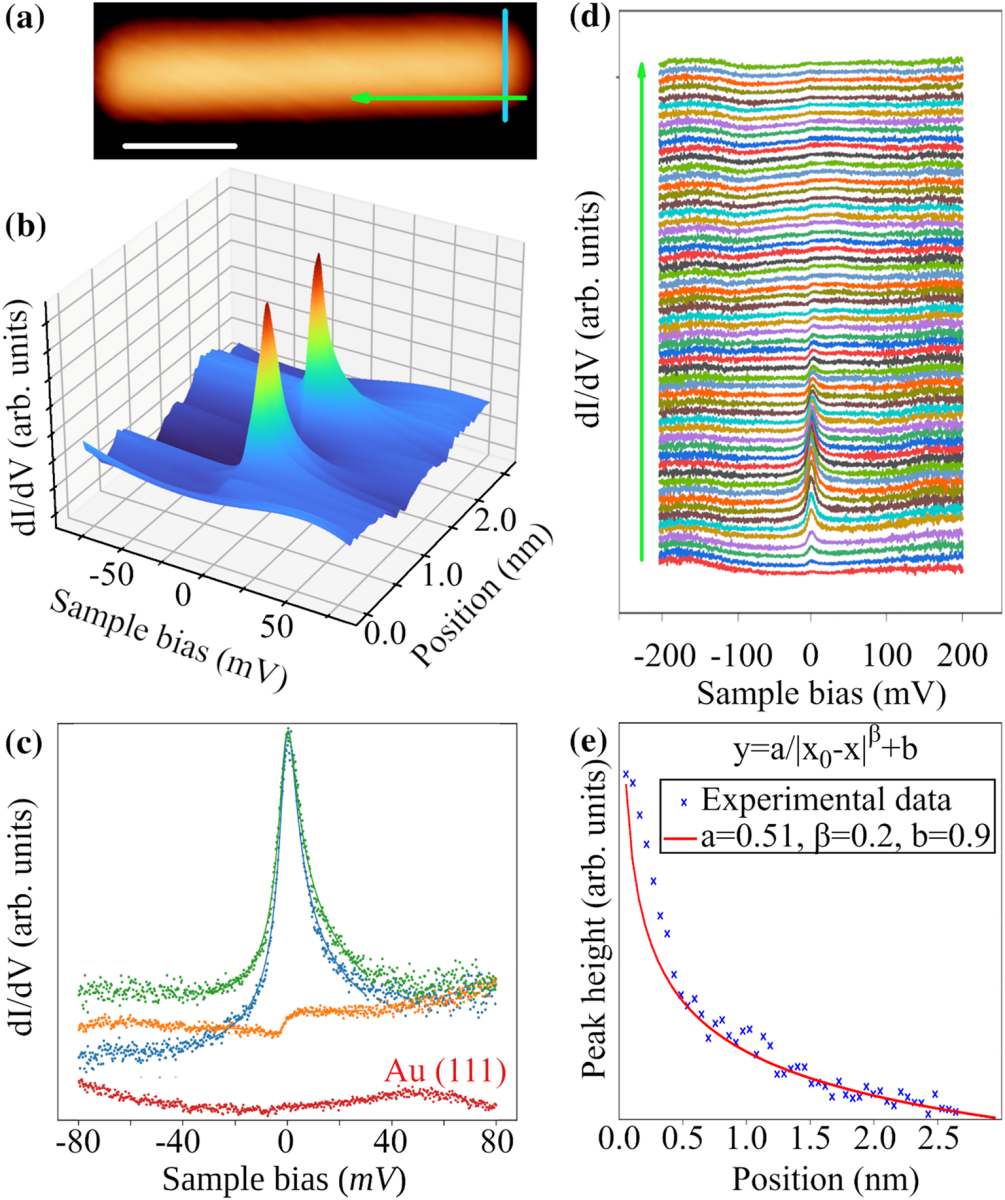
Spatial dependence of Kondo resonance. (**a**) Topographic STM image showing line scans of STS along and across GNR in green and blue colors respectively. The experimental parameters are − 500 mV, − 100 pA and 1.8 K for tunneling bias, current and sample temperature respectively. The scale bar is 2 nm. (**b**) dI/dV profile across the ribbon showing two Kondo resonances at the edge of the zigzag termini. (**c**) Comparison between the shape of Kondo resonances at both terminus peripheries (blue, green) and at the center (orange). Although the peak intensity drops sharply toward the center of the ribbon, it is still visible all the way between the magnetic centers. This indicates incomplete screening of magnetic moment and a possible spin coupling at each side. (**d**) dI/dV spectra showing the behavior of Kondo resonance along the ribbon edge. The red curve at the bottom is taken at Au(111). The offset between curves corresponds to a distance of 0.64 Å, covering a total distance of 3.15 nm in total. The peak intensity is highest at the zigzag terminus and gradually decays with broaden asymmetric shape toward the opposite terminus. (**e**) Profile of peak intensity along the nanoribbon edge shows delocalized Kondo resonance that persist to 2.5 nm away from zigzag terminus.

Next, we address the long-range spatial variation of Kondo resonance. The line profile of dI/dV spectra across the GNR terminus are shown in Fig. 3a. Two separate Kondo resonances are clearly identified at the edge of the ribbon. Away from the edges, the signal intensity drops off considerably but still remains visible at the central position. A comparison between the signal intensities at centers and edges are shown in Fig. 3b. These data suggest that the screening is partially incomplete and spin states of each side are weakly coupled. Away from the terminus, Kondo resonance gradually decays toward the opposite terminus but remains confined along the armchair sides. Figure 3c and Fig. 3d show the spatial variation of STS spectra and peak intensity along the armchair side of the GNR, respectively. From these profiles, we determine that the most appropriate location for the magnetic centers would be at the edge of the zigzag terminus^37^. The fact that Kondo resonance is not detected inside the GNR indicates no appreciable interaction effects from bulk π electrons and therefore the screening is mainly due to π edge states^3^. The relatively large screening length is surprising, given the fact that interaction with Au(111) substrate introduces various dephasing effects. The origin of this behavior is not clear yet. However, one possible reason is the competition between edge state π electrons and the substrate to screen the magnetic moment. Nevertheless, the peak at zero bias would indicate a weakly coupled ribbon with the underlying substrate. As the resonance intensity is decaying away from the unpassivated site, this competition may indicate that the edge state electrons dominate screening channels while the substrate acts as a secondary screening channel. At 2.5 nm away from the unpassivated site, the substrate effects take over and the Kondo signal disappears. For this reason, the observed decay in the signal intensity is most likely due to the weak interaction with substrate electrons. Earlier work^38^ has shown that the decay length can be accounted for by fitting spin–spin correlations to an exponential function with asymptotic tail. Similarly, we qualitatively deduce the spin–spin correlations length by fitting the spatial variation of peak height to a spatially decaying function with asymptotic tail ( $\propto {\left|x-{x}_{0}\right|}^{-\beta }$. The parameter $\beta$ determines the range of interactions. The best fit to our experimental data gives a value of 0.2 to $\beta$, as illustrated in Fig. 3d, which indicates a strong long-range coupling that extends over the edge of the ribbon. Having such spin states in supported GNR is very promising for applications in one-dimensional spintronic devices. However, a buffer layer is required to decouple the ribbon from surface to increase the range of spin–spin correlations to the entire length.

**Figure 4:**
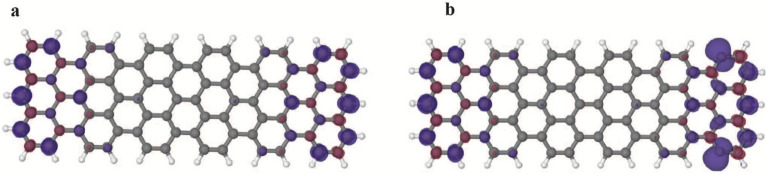
Spin densities for isolated ribbon. (**a**) The passivated and (**b**) the unpassivated GNR, cf. Figure 2a, surfaces are at 0.005 a_0_^−3^ (purple) and − 0.005 a_0_^−3^ (red). Further details are given in the supplementary information.

In order to understand the ground state properties and elucidate the spatial variation of Kondo resonance along the zigzag line, we have performed spin polarized DFT calculations (details are given in the supplementary information). We compare spin densities of isolated ribbons, i.e., without the Au (111) surface, for the fully passivated case with unpassivated ribbons, cf. Figure 2a. Also, two other types of ribbons with one unpassivated carbon on each side have been investigated (note that we only concentrate on the most likely possibilities with respect to experimental findings). Among the unpassivated ribbons considered, the one shown in Fig. 2a was found to be the most stable one. The comparison between spin densities of the passivated and unpassivated ribbons in gas phase is done for the following reasons: The spin density of the passivated ribbon is caused by two unpaired π electrons at the ends of the ribbons. The π electrons will interact rather strongly with the substrate in the adsorbed system. Therefore, we assume the spins in the π system will be quenched by the surface. Further, the unpaired electrons in the unpassivated ribbons are caused by the broken σ bonds and should have a much weaker interaction with the surface. This should enable us to roughly approximate the resulting spin density on the surface by the difference between the spin densities in gas phase, which are shown in Fig. 4. In Fig. 4a we show the spin density of the triplet state as the reference for the passivated ribbon. Note, that there is also a broken symmetry (BS) state with anti-ferromagnetic coupling between both ends of the ribbon. This state is only by a few meV below the triplet and shows more or less the same spin density, apart from a sign change between the ends. For convergence reasons we take the triplet state as reference in the following (see supplementary information for further details). For the passivated ribbon in Fig. 4a, the spin density is mainly localized at the zigzag termini and the density is slightly higher in the middle of each terminus compared with the edges, as can be seen in Fig. 4a. For the unpassivated ribbon, the spin density of the quintet state is shown in Fig. 4b (again we do not refer to the BS states, cf. supplementary information). Here, we observe a localization of the additional spin density mainly at the unpassivated sites of the ribbon (see Fig. 4b. These findings are not only valid for the hexamer, i.e., a ribbon formed from 3 DBBA precursors as shown in Fig. 4, but also for longer ribbons with up to 14 repeat units, i.e., formed from 7 DBBA precursors (isosurfaces of the computed spin densities are given in the supplementary information). To sum up, our results illustrate that delocalized spin states emerge as a result of de-hydrogenated sites near the GNR termini. The origin of the delocalized nature of the spin states is not clear yet, however, it may be related to open shell structure in the π bands of edge states.

Strong Kondo resonance emerges at the zigzag termini as a result of un-passivated atomic sites. Notably, Kondo peaks are most intense at each end atoms of zigzag chain, thus giving direct evidence to the presence of de-hydrogenated atoms at these locations. Interestingly, the resonances appear in pairs and vary considerably with a nonvanishing intensity along the line profile of the two magnetic centers suggesting more complex spin structure across the zigzag region. This behavior suggest that spin states are weakly coupled across the ribbon with a possible magnetic ordering at each side. The weak coupling at the zigzag terminus may lead to intricate ground state, that is not simply spin polarized, but rather a product of complex competition between local microenvironment and the quantum dynamics of spin states. The relatively long range of Kondo resonance indicates inefficient screening of the edge π electrons due to their one-dimensional character. The gradual decay of Kondo resonance may be explained by a secondary dephasing channel of Au(111) substrate. These results are supported by DFT calculations, which suggest a formation of spin quintet ground state. The presence of strong delocalized Kondo resonance along the armchair side of supported ribbon suggests a possibility to induce magnetism in GNR through de-hydrogenation of atoms at the peripheries of the zigzag termini. In future studies, it would be of great interest to perform tunneling spectroscopy on decoupled GNR to test the magnetic ordering without the influence of metal substrate.

## Methods

High quality of GNRs are fabricated using the well-known two steps annealing procedures on Au(111). However, as we have used a commercially available 10,10′-dibromo-9,9′-bianthryl (DBBA) precursor, the purity is further enhanced by degassing the source materials for a few days at 80 ºC prior to the evaporation procedures. To obtain a sparse sample, the DBBA is evaporated for a few seconds onto atomically clean Au(111) substrate which was held at 150 °C during the evaporation process. The growth process of GNR is initiated by annealing the sample for 15 min at 200 °C and at 350 °C. Finally, the sample is left to spontaneously cool down to room temperature before mounting into the STM chamber. In post annealing process we utilize a thermo-catalytic approach to abstract hydrogen atoms at most active sites of GNR. More specifically, we start by heating the GNR sample quickly from 4 K to a preheated stage at 350 °C. The temperature is then reduced within 15 min to 250 °C. The main annealing process was carried out for 12–24 h periods at 200–250 °C in UHV conditions. The samples were left to cool down with a rate of 2 °C/min prior to reaching 100 °C. The STM images haven’t been processed in anyway other than contrast enhancement, by WSxM software, for better visualizations^39^.

The single crystal of Au(111) substrate is cleaned by 5 cycles of sputter-anneal process. Prior to any further steps, the structure of the substrate was imaged by STM to ensure atomically clean Au(111) surface.

All imaging and spectroscopic experiments were carried out by using commercial UHV Joule–Thomson STM setup, which can be operated down to 1 K. High quality topographic images were obtained by scanning the samples at constant current. Typical tunneling parameters are 300 mV and 50 pA for sample bias and tunneling current respectively. The tunneling spectroscopies were performed by interrupting the feedback loop and ramping the bias voltages at various ranges. The tunneling spectra were obtained by using the standard lock-in techniques with modulations amplitudes ranging from 0.2 to 10 mV. The STM tips were made of freshly cut PtIr wires or electrochemically etched W wire. In both cases, the tips were sputtered for a few minutes using Ar^+^ plasma to remove any attached residues. Finally, the tips are cleaned in ultra-high vacuum by controlled crashing into a freshly cleaned Au (111) or Ag (111) at 100 V. From STS measurements on atomically clean metal surfaces, we consider the tips to be clean, if the spectra are featureless near zero bias with proper tunneling characteristics of substrate surface states as well as field emission resonances. The tunneling spectra were taken on clean Au (111) surface before and after any acquired data on the GNR to avoid any convolution with tip states. We have acquired a large number of spectra on several GNRs, all showing reproducible behavior for crystallographically equivalent locations. For reliable drift-free temperature dependent measurements, the system is left to thermalize at each temperature for 6 h before acquiring any tunneling spectra.

A MATLAB code is implemented to handle the deconvolution of Kondo resonance from instrumental and thermal broadening. In addition, the intrinsic HWHM is determined at each position by fitting the spectra to Fano-Frota line shape.

### Supplementary Information

Supplementary Information.

## Data Availability

The datasets generated during and/or analysed during the current study are available from the corresponding author on reasonable request.
