# Supplementary material for: Delocalized spin states at zigzag termini of armchair graphene nanoribbon
Source: Sci Rep. 2024 May 21;14:11641. doi: 10.1038/s41598-024-62624-9 (PMC11109170; doi:10.1038/s41598-024-62624-9)
Supplement: Supplementary file 1 — Supplementary Information. [file 41598_2024_62624_MOESM1_ESM.docx]

**Supplementary information**

Delocalized spin states at Zigzag Termini of Armchair Graphene Nanoribbon

Stefan Šćepanović^1, 2^, Amina Kimouche^3^, Jovan Mirković^2^, Gehad Sadiek^4^, Tillmann Klamroth^5^ and Abdou Hassanien^1, *^

*^1^Jozef Stefan Institute, 39 Jamova, Ljubljana, 1000,Slovenia*

*^2^ Faculty of Sciences, University of Montenegro, Podgorica, 81000, Montenegro*

*^3^Department of Physics and Astronomy, University of Potsdam, Potsdam, 14476, Germany*

*^4^* *Department of Applied Physics and Astronomy, University of Sharjah, Sharjah, 27272, UAE*

*^5^Institute of Chemistry, University of Potsdam, Potsdam, 14476, Germany*

*Email: [Abdou.Hassanien@ijs.si](mailto:Abdou.Hassanien@ijs.si)


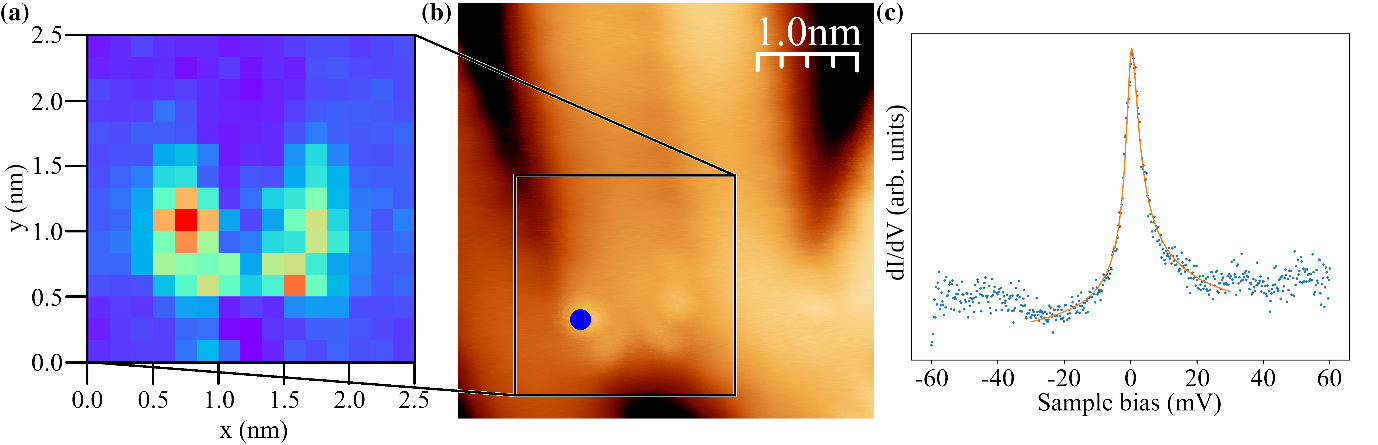


Figure S1. (a) Kondo map at the zigzag terminus of GNR shown (b). The tunneling parameters are -300mV and -50 PA. The locations of maximum signal intensities are obtained along the edge displaying a horseshoe shape. (c) Typical example of Kondo resonance taken at the blue dot in (b). The orange curve is a Fano-Frota fit to the experimental data. The fitting takes into the deconvolution from thermal broadening and lock-in modulation signals to obtain the intrinsic values of HWHM. All measurements were taken at 4.2K.


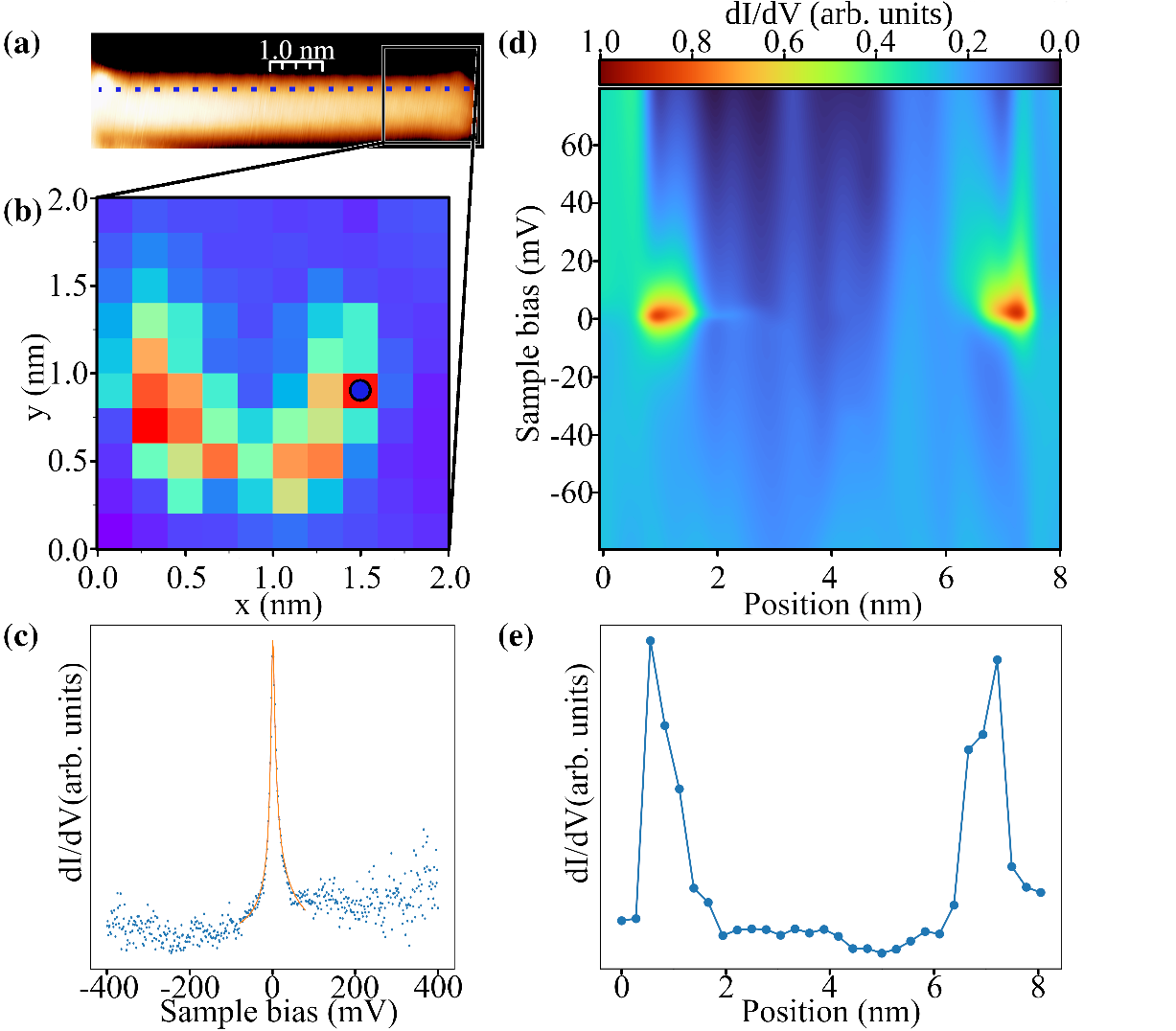


Figure S2. (a) Topographic map of a single GNR. (b) shows the zero bias dI/dV map of the GNR end. A well pronounced signal of Kondo resonance is marked with a blue circle and the appropriate dI/dV spectra are shown in (c) with a fitting to a Fano-Frota line shape (orange line) after deconvolution from thermal broadening and lock-in voltage modulation. (d) Line profile of dI/dV spectra along blue dotted line in (a) showing a well pronounced resonances at the edge of GNR. (e) The profile of signal intensity at zero bias displaying maximum well pronounced resonances at the edges. Measurements were taken at 4.2K.


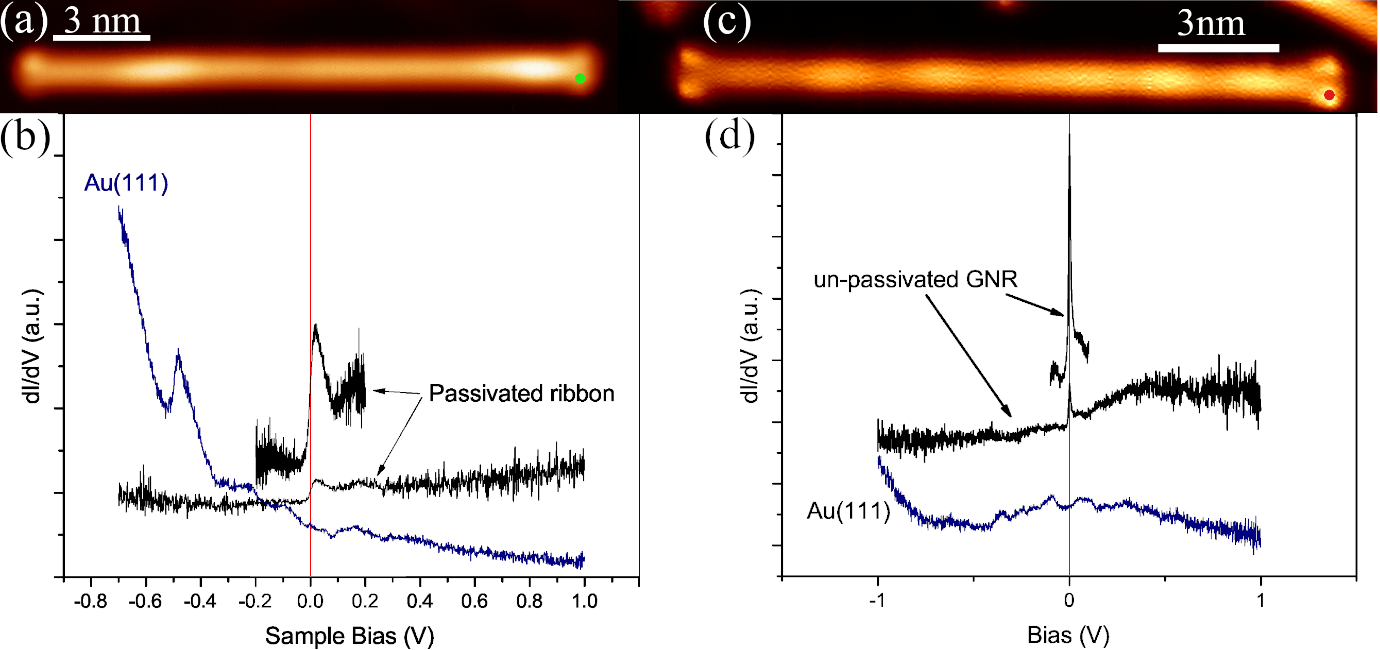


Figure S3. Electronic properties of termini before and after annealing process. (a) Constant height dI/dV map at zero bias of passivated GNR. (b) STS at marked location in green shown the edge state electrons with peak around 20 mV. (C) Constant height dI/dV map at zero bias of unpassivated GNR. (d) STS at marked location in red showing strong Kondo resonance at zero bias. Measurements were taken at 4.2K.

## Theoretical calculations

All DFT calculations were done using the Gaussian16^2^ program package with density functionals and basis sets as implemented therein. All results presented in the following are obtained for fully optimized structures of the GNRs in gas phase. By these DFT calculations we want to rationalize the GNR spin densities caused by hydrogen abstraction using the free
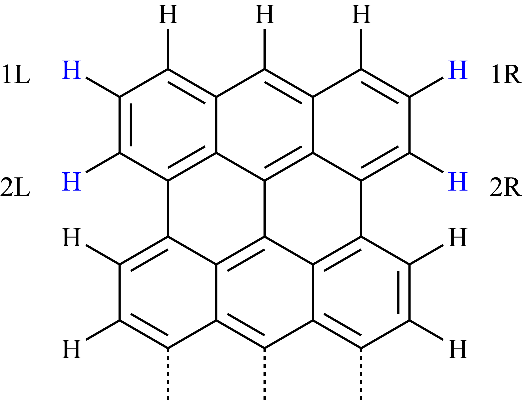
 standing GNR, i.e., without the supporting Au(111) surface.

Figure S4: The hydrogen atoms considered for abstraction on one end of the GNR are shown in blue alongside with their labels (1L,2L,1R,2R). One hydrogen on each side is removed, which leads to the 1L-1R, 1L-2R and 2L-2R structures. Note, 2L-1R is equivalent to 1L-2R.

Therefore, the first task is to determine the most stable spin state of the free standing GNR. Here, we use a hexamer, i.e., a GNR formed from three DBBA units, as for instance done in Ref. (1). In the present study, we also mainly employ PBE0 with the 6-311G** and the 6-31G* basis set based on the findings in our previous calculations^1^. For both basis sets, the most stable pure spin state of the hexamer is a triplet for the PBE0 functional, i.e. on the PBE0/6-311G** level of theory the singlet is 0.794 eV above the triplet and the quintet 2.089 eV. For PBE0/6-31G*, we find 0.977 eV for the singlet and 2.088 eV for the quintet. There is also a broken symmetry state (BS) with anti-ferromagnetic (AFM) coupling between both ends of the ribbon. This state has about the same energy as the triplet, i.e. for PBE0/6-31G* the BS state 3.8 meV below the triplet and 3.3 meV for for PBE0/6-311G**. However, calculations for the unpassivated GNR were very hard to converge or did not converge at all in reasonable states for broken spin symmetries. Therefore, we take the triplet state as our starting point, because it also shows almost the same spin densities on the GNR apart from a change in sign between both ends (see Fig. S5 (a1) & (a2)). For PBE and M062x, i.e. one functional without HF-exchange and one functional with a larger fraction of HF-exchange, we find the same energetic order for the pure spin states using the 6-31G* basis set.

In Figure S4 the hydrogen atoms, which will be removed to form unpassivated carbons on one end of the GNR hexamer, are labeled. We consider the following hydrogen abstraction patterns in the following: 1L-1R, 1L-2R and 2L-2R. We tested the following spin states: quintet, singlet and a BS singlet, i.e., a state where we start the SCF calculation with different spin-up an spindown densities. Also, BS triplets were tested. These states proof to be hard to converge in the right “broken” spin symmetry, i.e. a spin density which corresponds to the AFM state of the passivated ribbon. Further, the energies of the BS triplets were again – if converged – very close to the quintet state. Therefore, we did not use the BS triplets for the systematic investigations. Apart from the BS triplet, the quintet state of the 2L-2R structure (2L-2R-Q) is the most stable on both levels of theory. The most stable triplet structure is 1L-1R-T, which is 0.20 eV above 2L-2R-Q for PBE0/6-311G** and 0.19 eV for PBE0/6-31G*. All broken symmetry singlet states are at least 0.75 eV higher in energy and will not be considered in the following.

(a1) passivated


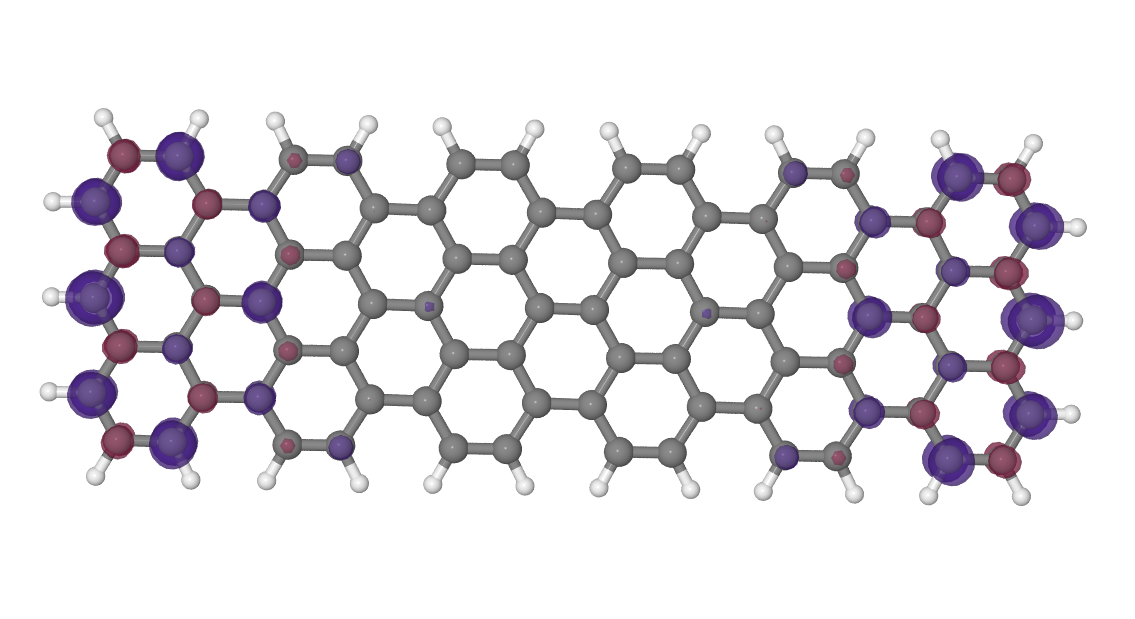


(a2) passivated BS


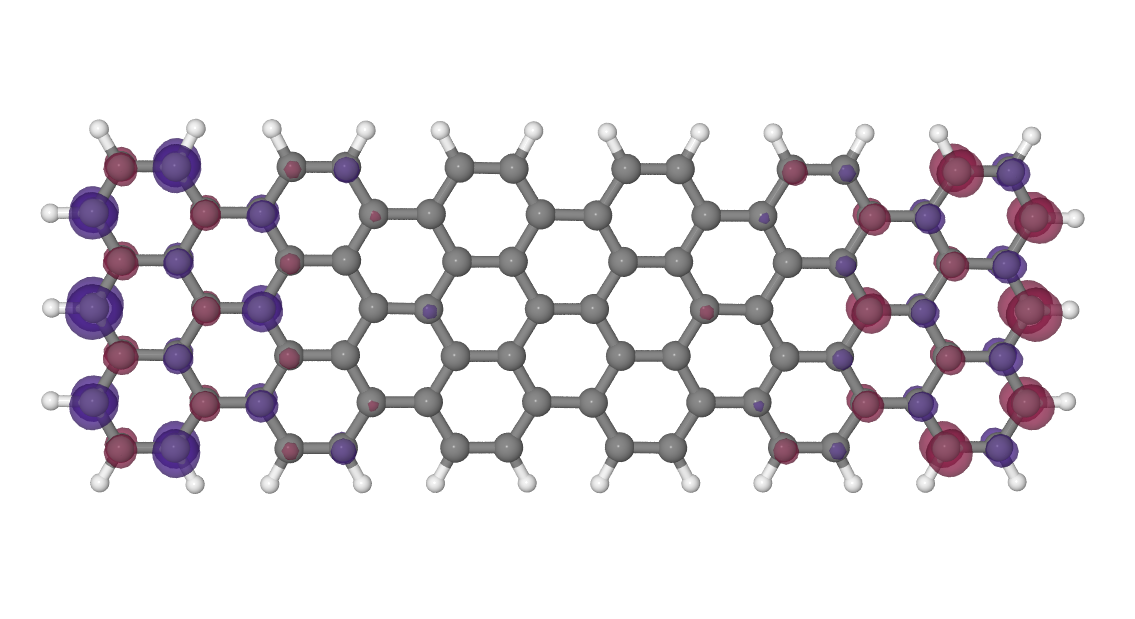


### (b) 2L-2R-Q


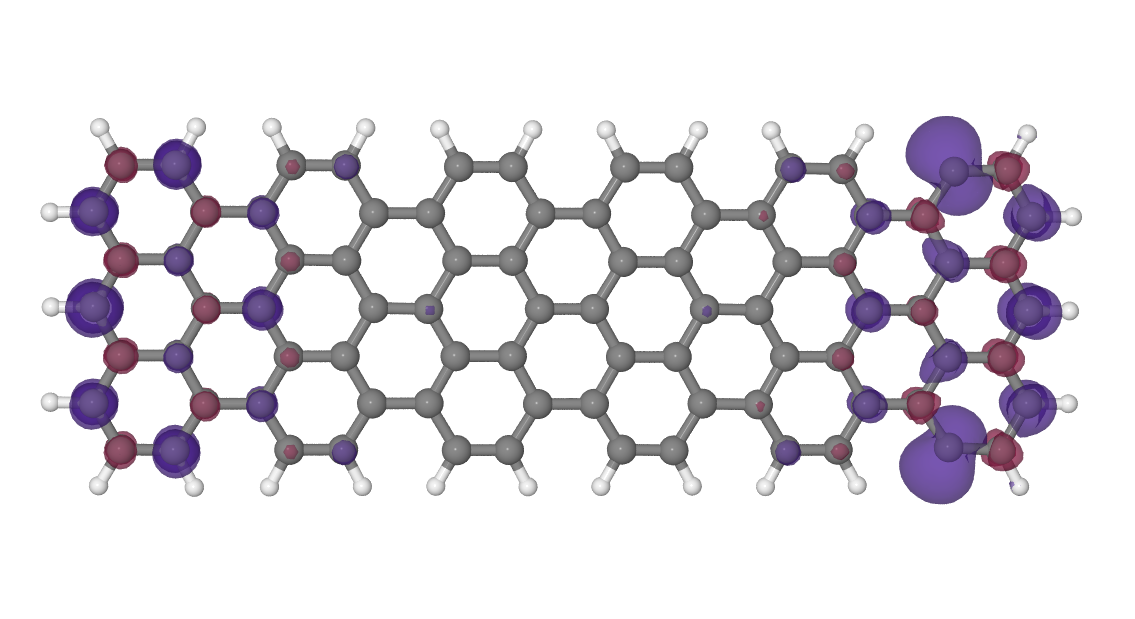


### (c) 1L-1R-T


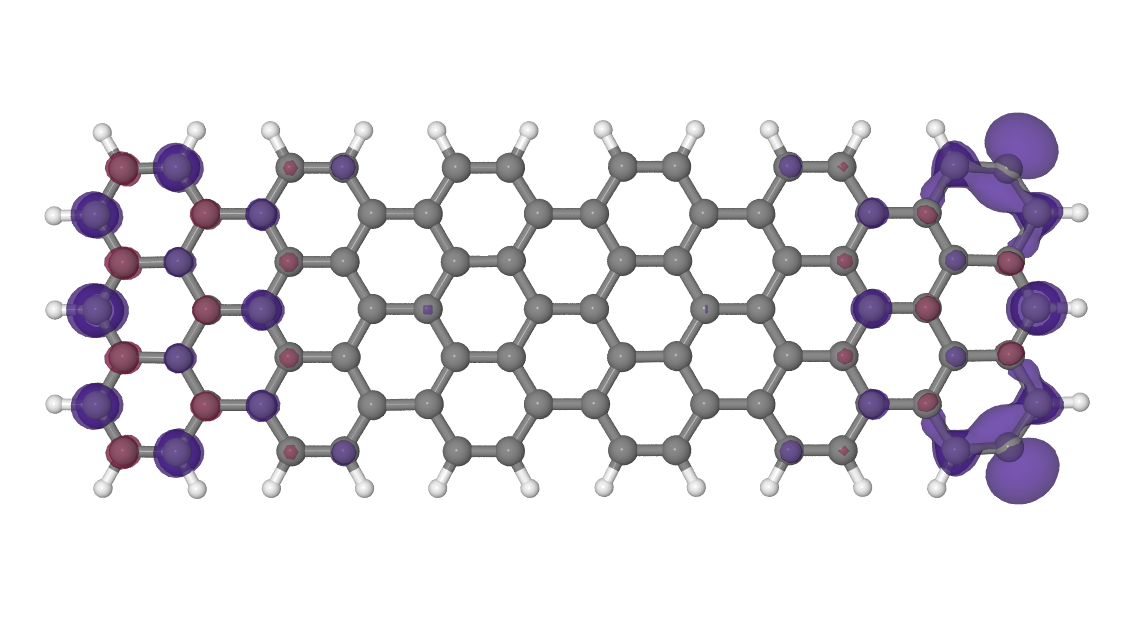


Figure S5. Spin densities for triplet (a1) and the broken symmetry state (a2) of the passivated GNR, (b) the 2L-2R-Q structure and (c) the 1L-1R-T structure at the PBE0/6-31G* level of theory for the hexamer, surfaces are at 0.005 a_0_^-3^ (purple) and -0.005 a_0_^-3^ (red).

The spin densities are calculated form the Gaussian16 log-files using molden^3^ and are written to cube-files. We use Jmol^4^ to visualize the spin densities from the cube-files. We present the spin densities for PBE0/6-31G* in the following, because the results are quite similar to PBE0/6-311G** and PBE0/6-31G* also allows the calculation of larger GNR oligomers, which will be compared to the hexamer. Figure S5 compares the spin densities of the passivated hexamer (a1) and (a2), the 2L2R-Q (b) and the 1L-1R-T (c) structure. One can see, that on the unpassivated end (right) of the hexamers the spin density for the 2L-2R-Q and the 1L-1R-T structure is located at outer C atoms of the GNR, i.e. unpassivated C atoms, while for the passivated hexamer the spin density is more located on the middle C atoms, i.e. the ones located on the zigzag ends of the GNR. On the passivated end of the GNR (left in Figure 4s), the 2L-2R-Q and the 1L-1R-T structure show nearly the same spin density as the passivated GNR.

We performed the same calculations for 2L-2R-Q and the 1L-1R-T structures with a higher number of repeat units, *n*, in the GNR on the PBE0/6-31G* level of theory. We chose even *n* up to 14, because the GNRs can be only assembled for even *n* starting from the precursor molecule DBBA. In all cases 2L-2R-Q is more stable than 1L-1R-T, with energy differences of 0.33 eV (*n* = 8), 0.33 eV (*n* = 10), 0.33 eV (*n* = 12) and 0.21 eV (*n* = 14). The spin densities for 2L-2R-Q and the 1L-1R-T structures with *n* = 14 are shown in Figure S6. Here, one can see, that the spin densities are nearly unchanged compared to the hexamer. Please note, that for the triplet state some changes on the passivated end of the GNR can be found for certain *n*, e.g., *n* = 12 (not shown).

### (a) 2L-2R-Q


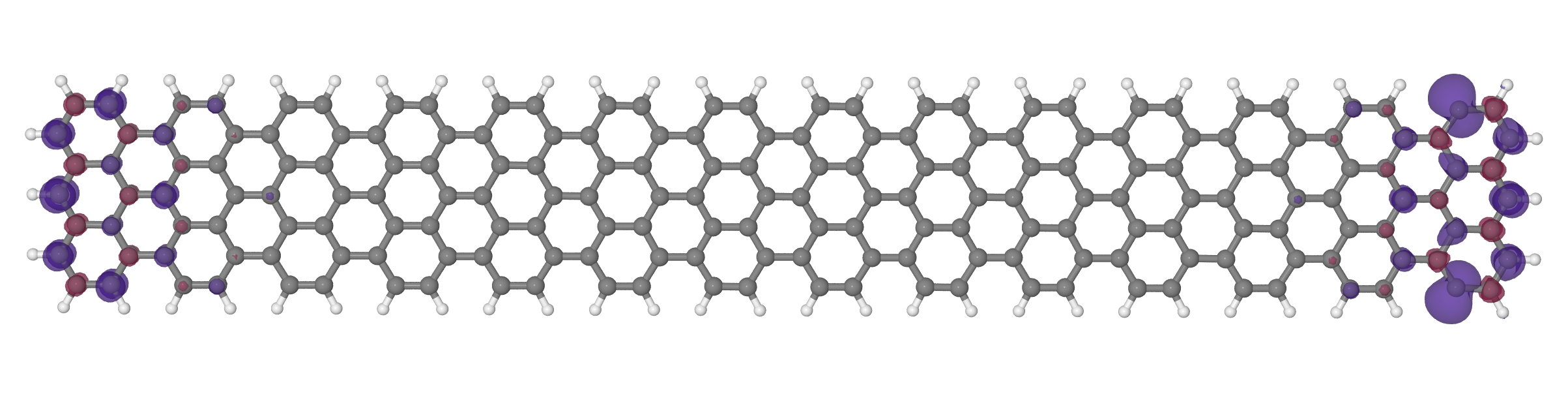


### (b) 1L-1R-T)


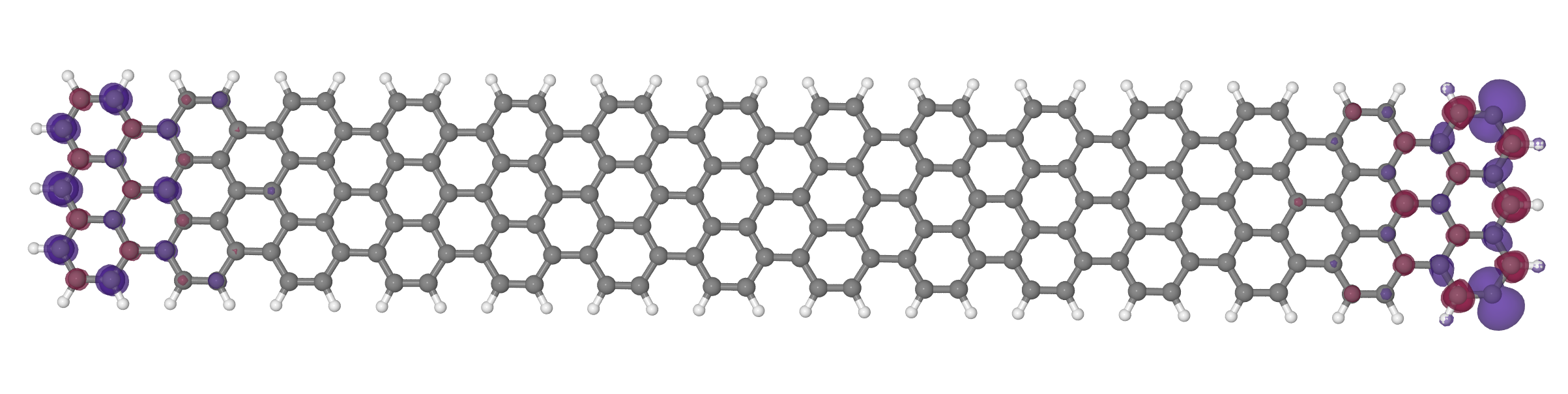


Figure S6. Spin densities for (a) 2L-2R-Q and (b) 1L-1R-T with *n* = 14 repeat units at the PBE0/6-31G* level of theory, surfaces are at 0.005 a_0_^-3^ (purple) and -0.005 a_0_^-3^ (red).

In summary, the calculations support the finding that hydrogen abstraction at one end of a GNR oligomer leads to a substantial spin density located at the unpassivated C-atoms, at least in the gas phase.

# REFERENCES

(1) Bronner, C.; Utecht, M.; Haase, A.; Saalfrank, P.; Klamroth, T.; Tegeder, P. Electronic structure changes during the surface-assisted formation of a graphene nanoribbon. J Chem Phys **140**, 024701 (2014)

(2) M. J. Frisch, G. W. Trucks, H. B. Schlegel, G. E. Scuseria, M. A. Robb, J. R. Cheeseman, G. Scalmani, V. Barone, G. A. Petersson, H. Nakatsuji, X. Li, M. Caricato, A. V. Marenich, J. Bloino, B. G. Janesko, R. Gomperts, B. Mennucci, H. P. Hratchian, J. V. Ortiz, A. F. Izmaylov, J. L. Sonnenberg, D. Williams-Young, F. Ding, F. Lipparini, F. Egidi, J. Goings, B. Peng, A. Petrone, T. Henderson, D. Ranasinghe, V. G. Zakrzewski, J. Gao, N. Rega, G. Zheng, W. Liang, M. Hada, M. Ehara, K. Toyota, R. Fukuda, J. Hasegawa, M. Ishida, T. Nakajima, Y. Honda, O. Kitao, H. Nakai, T. Vreven, K. Throssell, J. A. Montgomery, Jr., J. E. Peralta, F. Ogliaro, M. J. Bearpark, J. J. Heyd, E. N. Brothers, K. N. Kudin, V. N. Staroverov, T. A. Keith, R. Kobayashi, J. Normand, K. Raghavachari, A. P. Rendell, J. C. Burant, S. S. Iyengar, J. Tomasi, M. Cossi, J. M. Millam, M. Klene, C. Adamo, R. Cammi, J. W. Ochterski, R. L. Martin, K. Morokuma, O. Farkas, J. B. Foresman, and D. J. Fox. Gaussian˜16 Revision C.01, 2016. Gaussian Inc. Wallingford CT.

(3) Gijs Schaftenaar. Molden, version 6.7, 2020.

1. Jmol development team. Jmol, version 16.1.12, 2023-06-08.
